# Development and Quality Evaluation of Freeze-Dried Instant Green Smoothie Powder

**DOI:** 10.1155/2021/6634764

**Published:** 2021-01-28

**Authors:** S. H. B. Dilrukshi, H. P. S. Senarath

**Affiliations:** Department of Food Science & Technology, Faculty of Livestock, Fisheries & Nutrition, Wayamba University of Sri Lanka, Makandura, Gonawila (NWP), Sri Lanka

## Abstract

Fruits and vegetables are healthy food sources which contain nutrients and phytochemicals with health-promoting properties. The production of healthy and more convenient products can be contributed to increase the consumption of fruits and vegetables. In this study, a novel, instant food product which is more nutritive was developed using locally available fruits and green leafy vegetables and the physicochemical, microbial, and sensory qualities of the product were studied. The most acceptable formula for the fresh green smoothie was 28.6% green content with 71.4% fruit content. The developed green smoothie was freeze dried to obtain an instant green smoothie powder. The instant green smoothie powder was analyzed for proximate composition: protein (2.67 ± 0.00), fat (1.96 ± 0.001), moisture (4.82 ± 0.003), ash (1.22 ± 0.000), and fiber (28.57 ± 0.008). This instant powder contained 129.5 ppm vitamin C content and higher amount of minerals such as K (0.98 ± 0.12 mg/g), Ca (1.74 ± 0.03 mg/g), and Fe (0.04 ± 0.004 mg/g). The powder properties revealed that the powder has very good flowability (6.665 ± 2.354) according to the Carr Index and it has low cohesiveness (1.0713 ± 0.0264) according to the Hausner ratio. The solubility (94.71 ± 2.4253) of the powder complied with the SLS (668: 1984). The microbiological analysis revealed that this powder only contains 1 log CFU/ml of total plate count. This instant powder can be introduced as a more convenient and healthy choice for the consumers which has acceptable sensory properties, better microbiological stability, and very good powder properties.

## 1. Introduction

Fruits and vegetables are rich sources of dietary fiber, mineral, vitamin, and bioactive compounds which are extra nutritional constituents that naturally occur in slight quantities in foods [1, 2]. Scientific studies have confirmed a valuable association between consumption of adequate fruits and vegetables and prevention of chronic diseases [3]. But only 11.6%, 2.1%, and 3.5% of adults consumed the minimum daily recommended servings of vegetables, fruits, and both combined together, respectively, according to Jayawardena et al. [4]. Mainly, the current busy lifestyle does not allow the time required for the preparation of green leafy vegetables and it is one of the main obstacles for low consumption. Reflecting on this tendency, recently there was observed amplified demand for more innovative and convenient product with nutritional properties and consumer accessibility. Therefore, providing a novel product by using locally available fruits and green leafy vegetables for consumers is the most vital necessity in the present. In Sri Lanka, the postharvest loss of pineapple, avocado, and banana is 18%, 40%, and 30%, respectively [5]. Thus, the development of a fruit- and vegetable-based novel product with product diversification can be contributed to reduce the postharvest loss of fruits and vegetables in Sri Lanka.

Recently, smoothies have rapidly increased among the consumers' preferred choices of convenient ready-to-drink beverage, with suitable sensory properties in combination with nutritional benefits [6]. Daily consumption of green smoothies may enhance health quality of consumers. Depending on the serving size, smoothies can serve either as a main meal like breakfast or as a nutritious between-meal snack [7].

The limited shelf life is the main issue of the smoothie due to microbial spoilage and quality degradation. Therefore, increasing the shelf life of green smoothie by retaining the nutritional value and fresh-like quality is still a major challenge for the food industry [8, 9]. Freeze drying technique which removes the water by sublimation of ice from the frozen product is one of the most suitable long preservation technologies to preserve heat-sensitive nutrient in green smoothie. Production of freeze-dried instant powder from green smoothie is a novel process to increase the shelf life of the smoothies while keeping sensory and nutritional qualities. It is more important for the food industry.

The instant powder will provide more convenience for both manufacturers and consumers in packaging, handling, storage, and cost of transport and the easy preparation of the smoothie, respectively [10]. Today's trend is consuming nutritious food with more convenience which are safe in microbial point of view. Therefore, the present study was carried out to develop an instant green smoothie powder using locally available fruits and green leafy vegetables as a more convenient and healthy product for the consumers and to assess the quality parameters of instant green smoothie powder.

## 2. Materials and Methods

### 2.1. Development of the Product

#### 2.1.1. Materials

Fresh fruits and green leafy vegetables were used for this study. Fresh fruits such as banana (*Musa paradisiaca*), avocado (*Persea americana*), pineapple (*Ananas comosus*), ambarella (*Spondias dulcis*), green grapes (*Vitis vinifera*), and sweet orange (*Citrus sinensis*) and green leafy vegetables such as spinach (*Spinacia oleracea*), gotu kola (*Centella asiatica*), and broccoli (*Brassica oleracea*) were purchased from local supermarket in Pannala. Passion fruit leaves (*Passiflora edulis*) were obtained from the garden, Wayamba University of Sri Lanka. Maltodextrin was purchased from a food ingredient supplier in Colombo.

#### 2.1.2. Selection of the Best Formulation for Green Smoothie

Around 15 preliminary trials were done to select the most suitable varieties for this product. The best varieties of fruits and green leafy vegetables (banana, avocado, pineapple, spinach, gotu kola, and passion fruit) were selected based on the high concentration of nutrients, color, flavor, drinkable texture, and taste of the green smoothie. Sweet orange and water (1 : 1 ratio) were selected as the most suitable liquid for the green smoothie after four preliminary trials.

#### 2.1.3. Freeze Drying of the Smoothie

Sample preparation was mainly carried out at the Department of Food Science and Technology, Wayamba University of Sri Lanka. The prepared fresh green smoothie was freeze dried (Alpha 1-2 LD plus, Germany) for 45 to 48 hours with very low pressure and under a vacuum to obtain an instant powder. After freeze drying, the weight of the freeze-dried powder was measured and it was vacuum packed (DZ47-60G5, China) and stored under the refrigerated condition for further analysis.

#### 2.1.4. Preparation of Instant Green Smoothie

About 15 g of an instant green smoothie powder was dissolved in 100 ml of cool water. It can be consumed with a pinch of salt and honey or sugar with your preference.

### 2.2. Evaluation of Quality Parameters

#### 2.2.1. Sensory Evaluation

The acceptability of instant green smoothie powder was evaluated by conducting a consumer acceptability test [11] for 50 target consumers in the Food Preservation Laboratory, Wayamba University. Instant green smoothie samples (about 20 ml) were served in transparent glasses. Consumer acceptability test was conducted with a 7-point hedonic scale to evaluate attributes such as color, flavor, appearance, texture, taste, and overall acceptance with individual scores from 7 (like very much) to 1 (unlike).

#### 2.2.2. Physicochemical Evaluation

The pH of the sample was measured using a digital pH meter (STS 2100, USA) in triplicates for the fresh green smoothie sample and the instant green smoothie sample.

The total soluble solid content of the fresh green smoothie sample and the instant green smoothie samples was measured in triplicates using a handheld refractometer (Atago, Japan), having a range of 0-45 according to the method proposed by Castillejo et al. [12]. The total soluble solid of the sample was expressed as brix value (%).

Water activity of the fresh green smoothie sample and the instant green smoothie sample was determined using a water activity meter in triplicates.

#### 2.2.3. Evaluation of Powder Properties

Bulk density and tapped density were measured in duplicate according to the method proposed by Caliskan and Dirim [13]. The flowability and cohesiveness of the instant green smoothie powder were calculated using the method proposed by Caliskan and Dirim [13]. The measured value of bulk density and tapped density was used to calculate the flowability and cohesiveness of powder.

Solubility of the instant green smoothie was measured in a duplicate according to the method described in the Sri Lankan Standard (SLS) 668: 1984 with slight modifications. About 2.5 g of instant green smoothie powder was measured into a dried centrifuge tube. Water was added into the tube and shaken vigorously, and it was centrifuged. Then, the supernatant solution was pipetted out into dried, weighted moisture can. First, it was kept in a water bath, and then, it was oven dried (OF-22G, Korea). The weight was measured to calculate the solid in supernatant liquid, in g per ml. Finally, the weight of the residue was determined and solubility was calculated according to the following equation.

(1) $$\begin{array}{c} \text{Solubility}\left(\%\right)=\frac{\left[\left(m_{4}-m_{2}\right)–Y\left(m_{3}-m_{4}\right)\right]}{m_{1}}\times 100, \end{array}$$

where *m*_1_ is the weight of the sample (g), *m*_2_ is the weight of dried 50 ml centrifuge tube (g), *m*_3_ is the weight of the centrifuge tube with wet sediment (g), *m*_4_ is the weight of the centrifuge tube with dry sediment (g), and *Y* is the supernatant liquid, in g per ml.

#### 2.2.4. Evaluation of Proximate Composition

Moisture content, ash content, crude protein content, crude fiber content, and crude fat content of the instant green smoothie sample were measured using the AOAC (2000) method. Carbohydrate content was calculated by reducing the other compositions.

#### 2.2.5. Evaluation of Functional Properties

*(1) Total Antioxidant Capacity*. First, the crude methanolic extracts of the fresh green smoothie sample and the instant green smoothie sample were prepared and stored at -18°C till the analysis for a maximum of one week.

Total antioxidant capacity was determined according to the method described by Prieto et al. [14] with a slight modification. Briefly, 0.3 ml of crude extract was taken and 0.6 M sulphuric acid, 28 mM sodium phosphate, and 4 mM ammonium molybdate were added. Then, it was incubated at 95°C for 90 min. After the mixture had cooled to room temperature, the absorbance of each solution was measured at 695 nm spectrophotometrically (Evolution 201, China) against a blank. The antioxidant capacity was expressed as ascorbic acid equivalent (AAE).

*(2) Vitamin C Content*. The vitamin C content of the fresh green smoothie and the instant green smoothie was measured using 2,6-dicholorophenol indophenol visual titration method. About 10 ml of standard ascorbic acid solution was titrated with indophenol (V1). About 10 ml (V2) of the fresh green smoothie sample was titrated with indophenol (V3) in triplicates to determine the vitamin C content in the fresh green smoothie. About 1.5 g of instant green smoothie powder was dissolved in 10 ml (V2) of water, and it was used for the titration to determine the vitamin C content in instant green smoothie powder.

(2a) $$\begin{array}{c} \text{Dye equivalent V}1\text{ ml}=\frac{500}{1000}\times 10.00\text{ mg}. \end{array}$$

(2b) $$\begin{array}{c} \text{One milliliter of dye is equivalent to}\frac{500\times 10.00\text{ mg}}{1000\times \text{V}1}\text{ of ascorbic acid}. \end{array}$$

(3) $$\begin{array}{c} \text{Ascorbic acid content}=\frac{500\times 10.00\times \text{V}3\times 10^{3}\text{ ppm}}{1000\times \text{V}1\times \text{V}2}. \end{array}$$

#### 2.2.6. Microbiological Analysis

Total plate count was determined according to SLS 516: Part 01 instant green smoothie powder. Total yeast and mold count was determined according to SLS 516: Part 02 for instant green smoothie powder.

#### 2.2.7. Analysis of Mineral Content

The different mineral contents such as Ca, K, Mg, Fe, Zn, Na, and Cu were determined using an Atomic Absorption Spectrometer (ICE 3500, UK). First, the instant green smoothie powder was digested using a microwave digester (MARS 6, USA). Then, the standards were prepared and the mineral content was determined according to the method proposed by Paul et al. [15].

#### 2.2.8. Data Analysis

Sensory data was analyzed by the Kruskal-Wallis test at 95% significant level. For comparison of means, Tukey HSD was used and a significant difference was determined at *p* < 0.05.

## 3. Results and Discussion

### 3.1. Development of the Product

#### 3.1.1. Development of the Fresh Green Smoothie

Green smoothies are semiliquid beverage with smooth consistency, which consists of different fresh green leafy vegetables and fruits. Mainly, green leaves are used for the preparation of the fresh smoothie to obtain the green color and in view of their nutritional value.

Sweet orange juice was selected as the liquid medium due to the presence of citric acid, which can inhibit the growth of microorganisms. Therefore, it acts as a preservative; thus, this smoothie was prepared without using any preservative. During smoothie preparation, blending for 2 minutes is more important because it involves a breakdown of plant parenchyma, which leads to a dispersed solution consisting in a liquid phase with pectin and other soluble solids and a solid phase composed of insoluble solids with the cell wall [12].

About 26 g-30 g of Maltodextrin (MD) was added to prepare 100 ml (98 g) of fresh green smoothie. The addition of MD is important to minimize stickiness of particles during freeze drying. Maltodextrins are products of starch hydrolysis, consisting of D-glucose units linked mainly by *α* (1 → 4) glycosidic bond [16]. It is a generally recognized as safe (GRAS) compound which is used in the industry as a drying agent. The addition of the MD has more advantages such as increase solubility in the water, bulking, and film formation properties, playing a role in the reduction of oxygen permeability of wall matrix, tasteless, odorless, and functions including binding of flavor and fat [13].

#### 3.1.2. Development of the Instant Green Smoothie

Industrially, drying and dehydration processes have proven to be efficient and practical, for food preservation [1]. Freeze drying is normally recommended for drying of food which contains heat-sensitive compounds and antioxidant components [17]. Freeze drying is the dehydration process which removes water by sublimation of ice from the frozen product, and it is a long-term preservation of heat-sensitive food. Initially, the product is frozen solid, and then, it is exposed to a controlled temperature and reduced pressure environment [18]. Water in foodstuff is sublimed directly from a solid phase into a vapor phase at very low temperature in freeze drying [19]. Most of the characteristics of the fresh sample such as color, flavor, texture, nutrients, taste, appearance, chemical composition, shape, and biological activity slightly change in this drying technique [20]. Thus, freeze drying is one of the best methods for drying foods and for obtaining high-quality end product.

**Figure 1:**
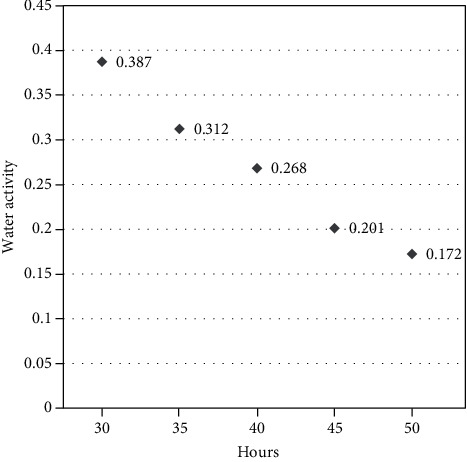
The graph of water activity of the instant green smoothie sample for different freeze drying times.

100 g of fresh green smoothie produced 25 g of instant green smoothie powder after freeze drying. Normally, freeze-dried powder has very low water activity: 0.197 in freeze-dried pumpkin puree powder [17]. Effective freeze-drying time was selected by comparing the measured water activity value (Figure 1) with this reference value. The sample should be freeze dried 48-50 hours to obtain the most suitable water activity value.

### 3.2. Quality Evaluation of Instant Green Smoothie

#### 3.2.1. Sensory Evaluation

**Figure 2:**
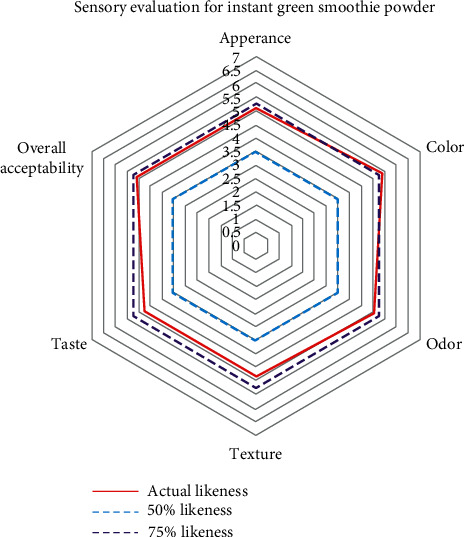
The radar chart corresponding to the sensory analysis of developed instant green smoothie powder. This chart indicates the mean rank of appearance, color, odor, texture, taste, aftertaste, and overall acceptability for the developed instant green smoothie powder. A red color line indicates the actual likeness, a purple color hachure line indicates the 75% likeness, and a blue hachure line indicates the 50% likeness for the developed instant green smoothie powder.

The developed instant green smoothie was evaluated for the sensory attributes of odor, color, appearance, taste, texture, and overall acceptability. Actual likeness of the instant green smoothie sample is at the margin of 75% likeness (Figure 2), when considering the odor, color, appearance, and overall acceptability. According to these results, taste and texture should be further developed to achieve the most acceptable level (75% likeness). The slightly bitter aftertaste could be reduced by adding a pinch of salt with sugar or honey according to consumer preference while dissolving the instant powder with cold water. A thickening agent could be added to improve the texture of the instant green smoothie to achieve 75% likeness for texture. The 50 semitrained panelists participated for sensory evaluation. Due to the fact that those panelists are semitrained, 50 were selected for this sensory evaluation. All panelists were in the age between 20 and 60.

#### 3.2.2. Physicochemical Properties of the Instant Green Smoothie Powder

Physicochemical properties of the fresh green smoothie sample and the instant green smoothie sample are summarized in Table 1. The total soluble solid and pH content for the instant green smoothie sample (Table 1) were measured after reconstituting the instant green smoothie powder. Moisture content and water activity for the instant green smoothie sample (Table 1) were measured in instant green smoothie powder.

The initial pH of the fresh (4.22 ± 0.020) and instant (4.44 ± 0.0385) green smoothie was not considerably different, and it is below pH 4.6. Therefore, *Clostridium botulinum* neither produce botulism toxin and germinate nor grow. Similarly, the initial pH of red fresh smoothie was 4.36 in the studies of Castillejo et al. [12]. MD and soluble fibers mainly contributed to the TSS content (22.27 ± 0.002) of the fresh green smoothie, and it was reduced into 12.33 ± 0.0028 after freeze drying. Castillejo et al. [12] reported that the red fresh smoothies have 8.37 and 7.07 total soluble solid. As observed, there was a high total soluble solid content in fresh and instant green smoothie comparing with those red fresh smoothies due to the addition of MD.

According to the results, the initial moisture content of fresh green smoothie was 88.12% and freeze drying removed 94.53% of water leading to a final moisture content of 4.82% in an instant green smoothie. This very low moisture content increases the shelf life of instant green smoothie powder. This result is slightly higher than the value of moisture content of freeze-dried pumpkin powder 3.93% and freeze-dried mango 4% as reported by Dirim and Çalışkan [17] and Marques et al. [21], respectively. Caliskan and Dirim [13] reported that increasing the MD concentration during freeze drying results in a significant decrease in moisture content of sumac extract powders due to an increase in solids in the feed and reduced amount of free water. The water activity of instant green smoothie powder was indicated as 0.197, and it is lower than the water activity level of freeze-dried pumpkin powder reported in Dirim and Çalışkan [17]. The water activity is a more important quality parameter for a dried product to determine the shelf stability. Very low water activity content confirmed the stability of the product for enzymatic activation, lipid oxidation, browning, and hydrolytic reactions [17].

#### 3.2.3. Proximate Composition of Instant Green Smoothie Powder

The proximate analysis was evaluated to find the available nutrient composition of the instant green smoothie powder, and it is a vital requirement before commercialization of a new product. This instant powder contained 1.22%, 1.96%, 2.67%, 4.82%, 28.57%, and 60.76% amount of ash, crude fat, crude protein, moisture, crude fiber, and carbohydrate content, respectively. The powder contained 271.36 cal/g of energy value. This result revealed that this powder contained high amounts of carbohydrate and crude fiber content. In a nutritional point of view, higher fiber content is more beneficial. This powder contained a lower amount of ash content when compared with the ash content of freeze-dried pumpkin powder (4.11 ± 0.08) reported in Caliskan and Dirim [13].

#### 3.2.4. Powder Properties of the Product

The quality parameters of the instant powder such as bulk density, Carr Index, and Hausner ratio were assessed to determine the powder properties. The measured powder properties revealed that it has 0.47 ± 0.0424 g/ml and 0.50 ± 0.0327 g/ml bulk density and tapped density, respectively. It is a higher value when compared with the bulk density of freeze-dried sumac extract 0.267-0.282 g/ml and bulk density of freeze-dried pumpkin powder 0.113 ± 0.0006 g/ml reported in Caliskan and Dirim [13] and Dirim and Çalışkan [17], respectively. This higher bulk density is important for storage and packaging of the powder because products with high bulk density are easy to store in small containers when compared to low bulk density products [22].

This instant powder has 6.665 ± 2.354% flowability and 1.0713 ± 0.0264 cohesiveness. The results of the powder properties revealed that this powder has very good flowability according to the Carr Index (CI) and very low cohesiveness according to the Hausner ratio (HR). CI is classified as very good (<15), good (15-20), fair (20-35), bad (35-45), and very bad (>45). HR is classified as low (<1.2), intermediate (1.2-1.4), and high (>1.4) [12]. The flowability of a powder is a measure of the free-flow characteristics. Proper flow of powder is very important for manufacturers and consumers for handling, packaging, measuring, transportation, bag filling and emptying, storage, and selecting parameters for mixing and conditioning [13].

The solubility of the instant green smoothie powder was 94.71 ± 2.4253%. This complied with SLS 668: 1984. Similarly, the freeze-dried guava powder was found highly soluble (96%) compared with other drying methods according to [13]. Normally, solubility is better in freeze-dried powder than powder, which is produced using other drying methods. The solubility is the most important property when considering the reconstitute ability of powder with water. This powder shows a better solubility due to the addition of the MD. Without the addition of the MD, there was a more clumping nature of the powder, with 8-12% TSS content and the average solubility time was 2 minutes. But with the addition of the MD, there was a less clumping nature of the powder with 21-24% TSS content and average solubility time was reduced into 50 seconds. This result revealed that the addition of the MD could decrease the average solubility time. Similarly, Caliskan and Dirim [13] reported that increasing the MD amount decreases the solubility time of the sumac extract powder. Freeze-dried pumpkin powder has 16 ± 0.58 seconds of average soluble time that is a very low soluble time reported in Dirim and Çalışkan [17] compared with the soluble time of this instant powder.

#### 3.2.5. Functional Properties of Green Smoothie Powder

The fresh green smoothie and the instant green smoothie sample contained vitamin C content of 148 ppm and 129.5 ppm, respectively. A slight reduction of vitamin C (12.5%) was observed after freeze drying. The reduction of vitamin C is lower than the vitamin C loss of freeze-dried pumpkin puree (18%) reported in Dirim and Çalışkan [17]. The loss of vitamin C was also found to be less than freeze-dried guava (37.47%) and pineapple (27.31%) [21], and it was higher than the mango (3.05%) and papaya (6.91%) [21]. The vitamin C losses can be due to not only freeze drying but also the operations before drying such as cutting, slicing, blending, and freezing. Dirim and Çalışkan [17] reported that the vitamin C reduction for freeze-dried fruits is considerably smaller when compared to the vitamin C losses caused by other drying methods due to the low temperatures and to the use of vacuum in the process.

An antioxidant is a major bioactive compound present in fruits and vegetables. The developed fresh green smoothie contained 121.37 ± 0.91 ascorbic acid equivalents mg/g of antioxidant capacity, and the instant green smoothie sample contained 107.25 ± 5.32 ascorbic acid equivalents mg/g of antioxidant capacity. The reduction of the antioxidant capacity was low (14.12%) during freeze drying. Thus, results revealed that the freeze drying process did not influence the nutritional value of the final product. 100 ml (15 g of instant green smoothie powder) of green smoothie sample covers 32.3% of the RNIs (adults) for vitamin C content.

#### 3.2.6. Evaluation of Microbial Stability

The unit operations of the smoothie processing, which includes several injury stresses such as peeling, cutting, shredding or blending, greatly raise the risk of microbial expansion [23]. But this instant powder has better microbial stability due to freeze drying. Fresh green smoothie contains 6.99 log CFU/ml and 8.255 log CFU/ml of total plate count and total yeast and mold count, respectively. But the instant green smoothie only contains 1 log CFU/ml of the total plate count. The water content of fresh green smoothie was reduced to a very low amount after freeze drying, thus not suitable to survive microorganisms. The reduction of the total plate count and total yeast and mold count was 85.6% (5.99 log units) and 100%, respectively, due to freeze drying. It is a higher value when compared with moderate heat and pulsed electric field treatments and mild thermal preservation methods. Because these treatments were reduced, total bacteria count was 1.9 and 1.8 log_10_ CFU/ml, respectively [24].

#### 3.2.7. Evaluation of the Mineral Content

100 ml of instant green smoothie sample provides the considerable amounts of K, Ca, Mg, Fe, Zn, and Na (Table 2). Therefore, 100 ml (15 g of instant green smoothie powder) of green smoothie sample covers 4%, 0.42%, 3%, 7.5%, 2%, 2%, and 49% of the Reference Nutrient Intake (RNI) (adults) for Ca, K, Mg, Fe, Zn, Na, and Cu, respectively.

## 4. Conclusion

An instant green smoothie powder contained locally available fruits and green leafy vegetables in ratios of 28% and 71%, respectively. It was developed as a novel product using the freeze-drying technique, without using any preservatives and sugar except MD as a drying agent.

The overall sensory acceptability of the product achieves 75% likeness level. This powder contained very low moisture content and very low water activity level, which can increase the shelf life of the product, and the product has a better microbial stability. Vitamin C loss and antioxidant reduction are 12.5% and 14.12%, respectively. This product can be used as a good fiber source due to its higher amount of crude fiber content. Therefore, the freeze-drying process did not influence the nutritional value of the final product. About 94% of solubility confirmed that it has a better reconstitute ability as an instant powder. Accordingly, this instant green smoothie powder can be introduced as a more convenient and healthy choice for the consumers due to the better quality of this powder.

## Figures and Tables

**Table 1:** Physiochemical properties (pH, water activity, total soluble solid, and moisture content) of fresh green smoothie and instant green smoothie sample.

Parameter	Fresh green smoothie sample (mean ± SD)	Instant green smoothie sample (mean ± SD)
pH	4.22 ± 0.020	4.44 ± 0.0385
Water activity	0.863 ± 0.0115	0.172 ± 0.0125
Total soluble solid (%)	22.27 ± 0.002	12.33 ± 0.0028
Moisture content (%)	88.12 ± 0.002	4.82 ± 0.020

Results are presented as mean ± standard deviation of three independent experiments.

**Table 2:** Mineral content (K, Fe, Zn, Mg, Ca, Na, and Cu) of the instant green smoothie sample and the provided mineral amount from 100 ml of instant smoothie sample (mg). Results are presented as mean ± standard deviation of two independent experiments.

Mineral	Quantity (mg/g)	Provided quantity from 100 ml of instant smoothie sample (mg/g)
K	0.98895 ± 0.12	14.83425
Fe	0.0435 ± 0.004	0.6525
Zn	0.0113 ± 0.003	0.1695
Mg	0.5138 ± 0.012	7.707
Ca	1.74225 ± 0.031	26.13375
Na	1.9049 ± 0.148	28.5735
Cu	0.03885 ± 0.03	0.58275

Results are presented as mean ± standard deviation of two independent experiments.

## Data Availability

The data used and/or analyzed in the study are available from the corresponding author on reasonable request.
